# Effects of Duration of Calcium Propionate Supplementation in Lambs Finished with Supplemental Zilpaterol Hydrochloride: Productive Performance, Carcass Characteristics, and Meat Quality

**DOI:** 10.3390/ani13193113

**Published:** 2023-10-06

**Authors:** Octavio Carrillo-Muro, Alejandro Rivera-Villegas, Pedro Hernandez-Briano, Marco Antonio Lopez-Carlos, Alejandro Plascencia

**Affiliations:** 1 Unidad Académica de Medicina Veterinaria y Zootecnia, Universidad Autónoma de Zacatecas, General Enrique Estrada 98500, Mexico; octavio_cm@uaz.edu.mx (O.C.-M.); pedro.hernandez@uaz.edu.mx (P.H.-B.); lopcarmarco@uaz.edu.mx (M.A.L.-C.); 2 Facultad de Medicina Veterinaria y Zootecnia, Universidad Autónoma de Sinaloa, Culiacan 80260, Mexico; alejandroplascencia@uas.edu.mx

**Keywords:** crossbred lambs, gluconeogenic precursors, small ruminant, β-adrenergic agonists

## Abstract

**Simple Summary:**

The finishing phase of lambs presents reduced efficiency and growth rates, due to a higher proportion of gain being fat rather than muscle. Hence, the primary objective during finishing is to increase the proportion of muscle while decreasing fat content. As an alternative, in this study the lambs were supplemented with calcium propionate (CaPr) and then finished with zilpaterol hydrochloride (ZH). Observations indicated that including CaPr for 28 d before slaughter improved the response to ZH supplementation in terms of feed efficiency, growth rate, carcass weight, and certain whole cuts.

**Abstract:**

Forty-five male non-castrated crossbred Dorper lambs (40.17 ± 0.35 kg body weight, BW) were employed in a completely randomized design with five treatments to investigate the effects of the duration of calcium propionate (CaPr) supplementation (10 g CaPr/lamb/d for 0, 14, 28, or 42 d before slaughter) on lambs finished with zilpaterol hydrochloride (ZH, 7.2 mg/lamb/d for a fixed period of 28 d before slaughter) regarding their productive performance, carcass characteristics, and meat quality. Treatments consisted of the following: (1) No additives (CTL), (2) 0 days on CaPr plus 28 d on ZH, (3) 14 days on CaPr plus 28 d on ZH, (4) 28 days on CaPr plus 28 d on ZH, and (5) 42 days on CaPr plus 28 d on ZH. When compared with CTL, ZH lambs exhibited a similar average daily gain (ADG) but had lower dry matter intake (DMI), leading to increased feed efficiency. Supplementing with ZH alone did not affect carcass traits, visceral mass, whole cuts, or meat quality. Lambs that received both CaPr 28 d and ZH exhibited quadratic increases (*p* < 0.05) in final body weight (FBW), ADG, and dressing percentage (D%). These increases were optimal at estimated inclusion durations of 26 d for FBW, 30 for ADG, and 39 d for D%. The ADG:DMI ratio and the longissimus muscle area (LMA) both exhibited quadratic increases (*p* < 0.05). The optimal duration of CaPr supplementation for ADG:DMI ratio was found to be 28 d, while for LMA, it was 14 d. As the period of CaPr supplementation increased, there was a linear increase (*p* < 0.05) in hot carcass weight, leg circumference, and whole cuts of breast IMPS209 and shoulder IMPS207. Cook loss percent increased quadratically (*p* < 0.05), and was higher when CaPr was included for an estimated duration of 26 d. As the duration of CaPr supplementation increased, the purge loss percentage (PRL) also increased linearly (*p* < 0.05). In conclusion, including CaPr in the diet for a duration of 28 d in lambs improved the response to ZH supplementation on the productive performance, carcass weight, and some whole cuts. However, it can also have a negative effect on PRL%.

## 1. Introduction

In recent decades, research on feedlot ruminants has primarily focused on finding technologies that improve feed efficiency and growth rates during the fattening process [1,2]. The final phase of finishing is characterized by lower growth efficiency in lambs, mainly because their gain consists predominantly of fat rather than muscle [3]. Thus, one of the main objectives during finishing is to augment the proportion or weight of muscles while decreasing the fat content in the carcass [1,2]. An alternative approach to achieve this goal is through the use of growth promoters, such as gluconeogenic precursors or β-adrenergic agonists (β-AA).

It has been observed that the gluconeogenic precursor calcium propionate (CaPr) alters energy metabolism in two ways when supplemented in ruminant diets: firstly, by altering rumen fermentation through improvements in ruminal dry matter (DM) digestibility, thereby increasing the proportion of ruminal propionate, and decreasing methane production [4,5]. Another way is by improving insulin’s action on glucose metabolism [6], thereby promoting an increase in energy status through enhanced glucose synthesis via gluconeogenesis in the liver [7]. In this context, improvements in growth performance, feed efficiency, and muscle growth have been reported in finishing lambs supplemented with a daily dose of 10 g of CaPr/d [8]. Carrillo-Muro et al. [9], studying finishing diets for lambs, determined that a dose of 10 g of CaPr/lamb/d for 42 d led to the following increases: 13% dry matter intake (DMI), 28% average daily gain (ADG), 17% ADG:DMI ratio, 7% final body weight (FBW) and 4% empty body weight (EBW); in addition, cooling loss percent (CL%) was reduced by 13%, without impacting meat quality variables. Furthermore, the duration of CaPr supplementation selectively impacts the benefits in terms of growth and carcass traits. Carrillo-Muro et al. [10] assessed varying inclusion durations of 10 g of CaPr/lamb/d, noting the following durations and their corresponding maximal increments: 15 d for DMI (1%), 25 d for FBW (5%) and ADG (27%), 28 d for ADG:DMI ratio (25%), 24 d for hot carcass weight (9%, HCW), and 20 d for dressing percentage (4%, D%). However, the extended inclusion duration (42 d) led to an increase in fat thickness (30%, FT) and a reduction in the proportion of loin (22%). Conversely, it increased the weight of leg (10%) and rack (14%). This suggests that the surplus energy provided by CaPr is stored as fat, resulting in a decrease in the proportion of certain muscles.

Zilpaterol hydrochloride (ZH) is a β-AA. When administered at a rate of 4 to 8 mg/kg diet during the final 20–40 d of fattening, it enhances growth performance, dietary energy utilization efficiency, and carcass traits in lambs [11]. The advantages of using ZH during the finishing phase of fattening have primarily been attributed to alterations in the composition of tissue gain [2,12]. Receptors in muscle and fat are activated by ZH, leading to augmented lipolysis, reduced lipogenesis, and increased protein accumulation, either individually or in combination [13]. However, these alterations in muscle tissue could impact the meat quality of lambs supplemented with ZH [14]. In support of this, a reduction of 8% in cook loss percentage (VFA%) has been noted in lambs supplemented with ZH [15]. Likewise, lambs that were administered 0.15 mg ZH/kg body weight (BW) experienced a 10% increase in Warner−Bratzler shear force [16].

Given the mechanism of action of CaPr and ZH, their combination could be complementary. Based on this, we hypothesized that supplemental CaPr in lambs receiving ZH during the final phase of fattening can enhance the response to ZH supplementation in terms of productive performance and carcass characteristics. Furthermore, the magnitude of these effects may be linked to the duration of CaPr. Therefore, the objective of the present study was to investigate the impact of varying inclusion durations of CaPr (0, 14, 28, or 42 d before slaughter) at a daily dose of 10 g in lambs finished with supplemental ZH (received ZH 28 d plus 3 d ZH withdrawal prior to slaughter) on productive performance, carcass characteristics, and meat quality.

## 2. Materials and Methods

The experiment took place at the Small Ruminant Experimental Center and Meat Science and Technology Laboratory, both located within the Unidad Académica de Medicina Veterinaria y Zootecnia at the Universidad Autónoma de Zacatecas (UAMVZ-UAZ), in the state of Zacatecas, Mexico (north-central Mexico). Throughout the experiment (April to May 2023), the ambient air temperature averaged 26.2 °C, with a minimum of 9.4 °C and a maximum of 32 °C.

All experimental procedures involving the lambs were conducted in accordance with the guidelines of the approved Official Mexicans Standards: NOM-051-ZOO-1995; NOM-062-ZOO-1999; NOM-024-ZOO-1995; NOM-033-SAG/ZOO-2014 and NOM-EM-015-ZOO-2002. In addition, the experiment reported herein was approved by the Bioethics and Animal Welfare Committee of UAMVZ-UAZ, with protocol number 2023/04.

### 2.1. Animal Housing, Basal Diet, Management, and Feed Sampling

Forty-five male non-castrated crossbred Dorper lambs, with an average initial body weight (IBW) of 40.17 ± 0.35 kg and aged 6 months, were used. The lambs’ IBWs were recorded, and they were accommodated in 45 individual pens (1.5 × 1.5 m). Three weeks prior to the trial beginning, all lambs underwent health management, which included the following: (1) identification with a uniquely numbered ear tag; (2) intramuscularly vaccination against *Clostridium* spp. and *Pasteurella* spp. (Exgon 10, Chinoin Veterinary, Aguascalientes, Mexico); and (3) treatment for endoparasites (Closantel 5%, Chinoin Veterinary, Aguascalientes, Mexico) and ectoparasites (Doramectin 1%, Dectomax, Zoetis, Ciudad de Mexico, Mexico). The lambs had an adaptation of 21 d to both the facilities and the basal diet. The composition of ingredients and the chemical characteristics of the basal diet are presented in Table 1. The diet was formulated to meet or exceed the finishing lambs’ recommendations for nutrients [17]. Throughout the study, lambs had unrestricted access to both the basal diet and fresh water. Fresh feed was provided twice daily at 800 and 1600 h in a 40:60 ratio, respectively. Feed offered to each lamb was adjusted to minimize waste (5% of the previous day’s intake, as-fed). Feed bunks were visually assessed between 0740 and 0750 h each morning, and any remaining feed was collected and weighed for determination of dry matter intake (DMI). Adjustments in daily feed delivery were made at the afternoon feeding. Prior to the morning feeding, the lambs were individually weighed at the beginning of the experiment (IBW), at intermediate points (14, 28 d) and at the end of the experiment (42 d). Daily samples of the basal diet were collected and analyzed in triplicate for the following: (1) DM%, dried for 24 h at 100 °C in a forced air-drying oven; (2) crude protein (CP) (FP-528 LECO nitrogen analyzer) [18]; (3) neutral detergent fiber (NDF) (fiber Ankom analyzer); and (4) Ether extract (EE) (extractor of Ankom^xt15^).

### 2.2. Experimental Design and Treatments

**Figure 1:**
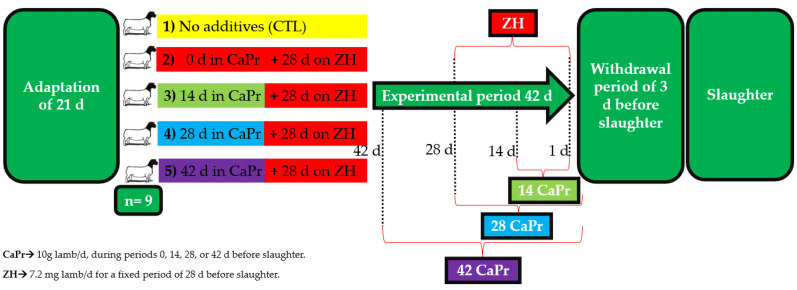
Completely randomized design and duration of calcium propionate (CaPr) supplementation in lambs finished with zilpaterol hydrochloride (ZH).

A completely randomized design with five treatments was employed to investigate the effects of CaPr administration during four treatment periods (0, 14, 28, or 42 d before slaughter) in lambs that were finished with ZH (for a fixed period of 28 d before slaughter). We randomly assigned the following treatments to the experimental units using a Microsoft^®^ Excel^®^ template: (1) no additives (CTL), (2) 0 days on CaPr plus 28 d on ZH, (3) 14 days on CaPr plus 28 d on ZH, (4) 28 days on CaPr plus 28 d on ZH, and (5) 42 days on CaPr plus 28 d on ZH. At the end of the fattening period, all lambs received a withdrawal period of 3 d before slaughter (Figure 1). The source of CaPr was Nuprocal^®^ (Nutryplus, Queretaro, Mexico), originating from the same batch, comprising 20% calcium and 69% acid propionic. The source of ZH was the same batch as the patented trademark Zilmax^®^ (MSD, Salud Animal Mexico, Estado de Mexico, Mexico). Individual CaPr (10 g/lamb/d) and ZH doses were weighed (precision balance, Pioneer-PX523, Ohaus Corp., Parsippany, NJ, USA); daily ZH dosage for feedlot lambs was 0.150 mg/kg average BW (47.8 kg), which essentially equates to 7.2 mg/lamb/d. The treatments were consistently administered by the same individual, and to prevent any influence on the lambs, the bags containing the doses were only identified with consecutive numbers (1 to 5), as seen in Figure 1. To ensure the treated group’s total intake, the doses were mixed with 100 g of the basal diet, offered in the morning. After consumption, the remaining portion of the diet was administered.

### 2.3. Productive Performance Calculus

Based on the data collected during the feeding trial, the following parameters were calculated: (1) ADG = [(FBW − IBW)/number of d on feed]; (2) Average DMI = (Feed offered—Feed refused), which was weighed and recorded daily; and (3) ADG:DMI ratio = (ADG/DMI).

### 2.4. Slaughter Procedure and Visceral Organ Mass Determination

At the conclusion of the 42 d trial period, the lambs underwent a fasting period (18 h) while having access to water. Pre-slaughter weights were recorded for subsequent calculations. During the slaughter process, non-carcass components, including skin, heart, lungs, liver, spleen, kidney, perirenal fat, and full and digesta-free gastrointestinal tract, were removed and weighed. EBW = (Pre-slaughter BW—Total non-carcass components weight). Visceral organ mass was expressed as g/kg of EBW.

### 2.5. Carcass Characteristics

The hot carcass weight (HCW) was determined prior to chilling (24 h at 4 °C). After the cooling process, the cold carcass weight (CCW) was measured, including the kidneys and internal fat. Carcass D% was calculated as = ([CCW/EBW] × 100), and the percentage of cooling loss (CL%) = ([HCW-CCW]/HCW) × 100. After a 24 h chilling period, the carcass dimensions were measured using a flexible tape measure, including carcass length, leg length, and chest circumference. Longissimus muscle area (LMA) and FT between the 12th and 13th ribs were assessed on days 0, 14, 28, and 42, employing an Aloka Prosound 2 instrument with a 3.5 MHz linear transducer.

### 2.6. Whole Cuts and Tissue Composition

The left sides of the carcasses were sectioned in accordance with the guidelines of the Institutional Meat Purchase Specifications (IMPS) and North American Meat Processors Association, to extract the (1) forequarter, which was further subdivided into the neck, shoulder IMPS206, shoulder IMPS207, rack IMPS204, breast IMPS209, ribs IMPS209A; and (2) hindquarter, comprising the loin IMPS231, leg IMPS233, and flank IMPS232 [19]. The weight of each cut was then recorded and expressed either in g/kg of EBW or as a percentage of the CCW. The carcasses were halved, and the left side was dissected. The tissue composition of the shoulder was determined via a physical dissection process to calculate the percentage of muscle, fat, and bone [20].

### 2.7. Meat Quality

The 9th to 10th rib section of the longissimus muscle (LM, approximately 500 g) from the right-half carcass was used for subsequent meat quality analysis (frozen at −20 °C).

Color measurements were taken from the surface of the LM exposed by the 12th/13th-rib cut, using a Minolta CR-400 spectrophotometer (Konica Minolta Sensing, Inc., Osaka, Japan). The configuration included an 8 mm aperture size, observer 10, D65 illuminant, and a blooming time of 1.5 s. The color coordinates, including luminosity (Hunter L* Value), redness (Hunter a* value), and yellowness (Hunter b* value), were measured after 24 h postmortem. The pH of the right LM at the 2nd lumbar vertebra (LM) was determined using a portable digital pH meter (Hanna Instruments, Model HI–9025).

The water-holding capacity percent (WHC%) was assessed following the methodology described by Grau and Hamm, as suggested by Tsai and Ockerman [21]. In summary, 300 mg of LM was enclosed with filter papers (Whatman #1), positioned between glass plates (15 × 15 cm), and subjected to a consistent pressure of 10 kg for 20 min. LM steaks (2 cm thick) acquired from between the 12th rib and L2 vertebrae were vacuum-sealed in plastic bags and frozen at −20 °C. After being stored for 14 d, the steaks were allowed to temper for 24 h at 4 °C, then gently blotted dry and weighed. One steak was sliced into dimensions of 15 mm × 15 mm × 30 mm, then suspended at 4 °C for 24 and 48 h, to calculate the percentage of purge loss (PRL%). Another steak was vacuum-sealed in a polyethylene bag and heated to 80 °C until the internal temperature reached 70 °C for determination of CKL%. The sealed plastic bag samples were placed individually and submerged in a water bath at 75 °C until they attained an internal temperature of 70 °C. Following the cooking process, the samples were cooled under running tap water, extracted from the packaging, gently blotted, and weighed. The WHC%, PRL%, and CKL% were expressed as percentages of weight loss compared to the initial weight. This was calculated as [(initial weight − final weight)/initial weight] × 100.

Steaks (2.54 cm thick) were thawed for 24 h at 4 °C. They were subsequently broiled on an electric grill, specifically the George Foreman Electronics (Model GR2120B), until they reached an internal temperature of 70 °C [22]. The internal temperature was monitored using a Kitchen thermometer (Model TP700). After the cooking process, the steaks were allowed to cool at 22 °C for 4 h. In due course, six cores, each measuring 1.27 cm, were extracted parallel to the muscle fiber using a mechanical coring device. These cores were then subjected to shearing using a Texture Analyzer (G-R Manufacturing, New York, NY, USA) equipped with a Warner−Bratzler knife, and peak shear force achieved was recorded. The crosshead speed was set to 200 cm min^–1^. The shear force measurements were then averaged and expressed in kg/cm^2^.

### 2.8. Statistical Analyses

The statistical analysis was conducted using SAS OnDemand free software. Normality assumptions were validated through the UNIVARIATE procedure. The data were analyzed using a completely randomized design with the GLM procedure. ADG, DMI, and ADG:DMI ratio were analyzed using the MIXED procedure for repeated measurements. Lambs and carcasses were considered as the experimental units for productive performance and meat characteristics. The CCW was introduced as a covariate for the analysis of carcass characteristics. When significant effects were detected, mean comparisons were carried out employing the Tukey method with the LSMEANS instruction.

The duration of CaPr supplementation was categorized into linear and quadratic orthogonal polynomials, involving four equally spaced levels, using the LSMEANS and ESTIMATE statements. In instances where quadratic polynomials exhibited significance, quadratic equations were computed utilizing the REG procedure. Significance was established when the *p*-value was ≤0.05, and a trend was considered if the *p*-value was >0.05 and ≤0.10.

## 3. Results

### 3.1. Productive Performance

Compared to CTL, ZH lambs exhibited similar ADG but a 28.5% reduction in DMI, leading to a notable 26.4% enhancement in feed efficiency. The reduction in DMI due to ZH supplementation persisted although to a lesser extent in the presence of CaPr. Lambs that received both ZH and CaPr exhibited a quadratic response (*p* = 0.04; Table 2) for FBW and ADG. Regression analysis revealed that the peak values were estimated at 26.4 d and 30.5 d, respectively. The ADG:DMI ratio displayed an increase with the inclusion for 28 d (quadratic trend, *p* = 0.09), while no significant effects were noted for DMI (*p* = 0.15). Remarkably, the inclusion of CaPr for 28 d led to enhancements (*p* < 0.05) in FBW (9.5 and 8.4%%), ADG (51.4 and 42.3%), and ADG:DMI ratio (76 and 29.4%) compared to the CTL group and the ZH-only group, respectively.

### 3.2. Ultrasound Measurements, Carcass Characteristics and Shoulder Composition

Supplementing with ZH alone did not exert any significant impact on carcass traits, visceral mass, whole cuts, or meat quality. However, when ZH-supplemented lambs also received CaPr, alterations in carcass traits were observed. Specifically, D% exhibited a quadratic response (*p* = 0.05; Table 3), with the maximum value estimated at 39.1 d of CaPr inclusion through regression analysis; additionally, LMA increased with a 14 d inclusion period (quadratic effect, *p* = 0.05). Likewise, as the duration of CaPr inclusion extended (linear effect, *p* < 0.05) there were increments in HCW, CCW, and leg circumference. No significant effects were detected in the remaining variables (*p* > 0.05). Lambs that received both CaPr and ZH displayed an increase (*p* > 0.05) of 11.3% in LMA compared to CTL and 10.4% compared to ZH. Lambs supplemented with CaPr for 28 d exhibited higher (*p* > 0.05) HCW and CCW compared to CTL and ZH lambs (*p* > 0.05), but the differences were not statistically significant when compared to lambs receiving CaPr for 14 and 42 d. Supplementing CaPr for any duration led to an increase (*p* > 0.05) of 9.9% in the D% relative to CTL and a 5% increase compared to ZH. There were no significant differences among the treatments (*p* > 0.05) for the other evaluated variables.

### 3.3. Visceral Organ Mass

Supplementing with ZH alone did not have any effect on visceral mass. The EBW showed a quadratic response (*p* = 0.03; Table 4), with the maximum value estimated at 54.9 d for the inclusion of CaPr according to regression analysis. Overall, the inclusion of CaPr led to an average increase (*p* < 0.05) in the EBW of 10.7% compared to the CTL and of 4.8% relative to ZH, without influencing visceral organ mass (*p* > 0.05).

### 3.4. Whole Cuts

Supplementing with ZH alone did not yield any significant effects on whole cuts. As the duration of CaPr supplementation increased, the values (expressed as g/kg of EBW or as percent of CCW) of breast IMPS209 (linear effect, *p* < 0.05, Table 5) and shoulder IMPS207 (linear trend, *p* = 0.09) also increased. No significant effects were observed for the remaining whole cuts (*p* > 0.05). Specifically, the inclusion of CaPr (duration of 14 to 28 d) increased (*p* < 0.05) the rack IMPS204 by 19% compared to ZH and led to a substantial 38% increase compared to CTL.

### 3.5. Meat Characteristics

Supplementing with ZH alone did not exert any discernible effects on meat characteristics. However, the CKL% value was higher (*p* > 0.05) in the treatments with CaPr compared to CTL and ZH alone. Within the duration period of CaPr supplementation, lambs expressed a quadratic response (*p* = 0.001; Table 6) in CKL%, with the maximum value estimated at 25.8 d according to the regression analysis. The duration of CaPr supplementation showed a linear effect on PRL% values, resulting in increased values at 24 h (trend, *p* = 0.06) and 48 h (*p* = 0.03). Moreover, PRL_48_% was higher in lambs that received CaPr supplementation for 28 and 42 d (*p* < 0.05). No significant differences were observed between the treatments regarding the rest of the evaluated meat variables (*p* > 0.05).

## 4. Discussion

### 4.1. Productive Performance

An increase in dietary energy consistently stimulates the improvement in the productive performance of finishing lambs [23,24,25,26]. When CaPr is supplemented alone, it undergoes hydrolysis within the acidic pH of the rumen, resulting in the formation of Ca^2+^ and propionic acid [7]. This process yields several effects at the rumen level, including (1) alteration of the volatile fatty acid (VFA) pattern [5]; (2) reduction in methane production; (3) improved digestibility of the DM; (4) enhanced fermentation efficiency [4]; and (5) improved responsiveness of insulin in glucose metabolism [6], which plays a role in both fat deposition and muscle growth. As a cumulative outcome of these mechanisms, there is a promotion of energy status achieved through the heightened synthesis of glucose via gluconeogenesis [7].

The findings of this study are consistent with those observed when CaPr was administered individually. In a similar vein, Carrillo-Muro et al. [10] determined the duration of CaPr supplementation in Dorper × Katahdin lambs (IBW 39.1 kg) with diets containing 72% grains, and observed maximum increases at 25 d for FBW (4.7%) and ADG (26.8%), and 28 d for ADG:DM ratio (25.8%). Interestingly, they also observed a peak in DMI (1.1%) at 15 d, with a subsequent reduction in the variable observed at the 42 d mark. However, in this study, it was observed that in the presence of ZH, the peak values were apparent within the range of 25 to 30 d and declined at 42 d; nevertheless, the observed increments surpassed 8.4% for FBW, 42.3% for ADG, and 29.4% for ADG:DMI ratio. Surprisingly, no discernible differences were noted in DMI. This observation aligns with the findings of Martínez-Aispuro et al. [8], yet it contrasts with the results reported by Carrillo-Muro et al. [9], who reported an increase in DMI. Both studies, however, corroborate that the inclusion of CaPr for 42 d in the finishing phase enhances FBW, ADG, and ADG:DMI ratio compared to not incorporating CaPr.

The use of β-AA enhances ADG and ADG:DMI ratio [2,27], stimulating protein synthesis in skeletal muscle and inhibiting its degradation. In adipose tissue, this supplement amplifies lipolysis [28]. In our study, the improvements in ADG and ADG:DMI ratio achieved by supplementing lambs with CaPr for 28 d in the presence of ZH surpassed those observed in previous reports when ZH was administered individually [2,12,29]. The literature states that ZH supplementation in finishing lamb diets results in ADG enhancements ranging from 20.1% to 40.6%, alongside ADG:DM ratio increases ranging from 16.5% to 43.3%. However, our research yielded even greater gains, revealing a 9.5% upsurge in FBW, a remarkable 51.4% escalation in ADG, and an impressive 76% increase in ADG:DMI ratio. Likewise, the decrease in DMI reported by other authors upon ZH supplementation (13.3% to 16.2%) [12,14,29,30] aligns with the findings of the present study (13.2%). This decrease in DMI, coupled with the heightened ADG:DMI ratio, likely signifies ZH’s direct influence on net protein retention, consequently impacting the growth of lean tissue [31]. This ultimately leads to a more efficient use of nutrients for ADG, resulting in substantial feed savings (13.2%) compared to CTL.

### 4.2. Ultrasound Measurements, Carcass Characteristics and Shoulder Composition

Piola-Junior et al. [26] mentioned that elevating the energy level in the diet of fattening lambs has positive effects on carcass characteristics, revealing a linear connection between ME and HCW (R² = 0.9), CCW (R² = 0.95), and D% (R² = 0.91). Furthermore, Moloney [32], while incorporating sodium propionate (0 or 40 g/kg DM) into the diet, pointed out that it alters the growth and composition of lamb carcasses. In addition, they observed a decrease in fat deposition, an increase in skeletal muscle growth, and a change in the acetate:propionate ratio in rumen fluid. This shift was attributed to increased protein accumulation to absorbed propionate, which is used for gluconeogenesis, thereby sparing amino acids for fueling protein synthesis.

A study conducted by Carrillo-Muro et al. [10] aimed to ascertain the duration of CaPr supplementation in lambs and found peak enhancements at 24 d for HCW and CCW, and 20 d for D%, but observed no effects on FT and LMA. All these variables were reduced at 42 d. Notably, in the current study, when CaPr was combined with ZH for the same duration, optimal values were observed in the range of 14 to 28 d and reduced at 42 d. Additionally, an increase in LMA was observed with CaPr supplementation for 14 d when combined with ZH. This alignment corresponds with the findings of Cifuentes-López et al. [33], who reported that sheep fed with CaPr for 42 d showed improvements in D% and LMA. Additionally, Carrillo-Muro et al. [10] reported an increase in HCW and CCW along with a lower CL. However, there was no significant effect on D%. Conversely, Martínez-Aispuro et al. [8] did not find any noticeable impact on FT and LMA in lambs over a 42 d period with CaPr. Furthermore, in lambs fed finishing diets with CaPr supplementation for 42 d, Lee-Rangel et al. [34] reported no discernible differences in HCW or LMA (CaPr vs. CTL).

The consistent response of increased LMA, HCW, CCW, and D% through ZH supplementation has been well established in finishing lambs within the feedlot setting [2,11,27]. The increase in LMA is mainly due to the greater ADG [35] and the growth in shoulder muscle [Partida et al., 2015]. The increase in D% by 3.9% with CaPr, observed by day 28 when combined with ZH, resulted in 2.2 kg more salable meat compared to ZH due to CaPr supplementation. This increase in D% coincides with several previous studies in which ZH was supplemented [3,36]. This outcome can be attributed to muscle hypertrophy caused by increased protein synthesis and reduced protein breakdown [37,38], leading to reduced fat [39], a parameter unaffected by any treatment in our study.

### 4.3. Visceral Organ Mass

The results of the present study showed maximum increases in EBW of 10.7% compared to CTL and 4.8% compared to ZH at 55 days. However, Carrillo-Muro et al. [10], investigating lambs supplemented over different periods, identified peak increases at 28 d, followed by a decline at 42 d. This suggests that these increases in EBW are attributable to the increase in energy availability offered by the lambs’ gluconeogenic precursors. Additionally, Carrillo-Muro et al. [9], when feeding CaPr for 42 d to finishing lambs, reported that EBW increased, while with ZH supplementation in lambs, Rivera-Villegas et al. [2] noted that EBW increased by 4.6%.

The impact of CaPr and ZH on the visceral organ mass has garnered limited attention. However, Carrillo-Muro et al. [10], investigating fattening lambs, did not observe effects on organ weights. In contrast, Cifuentes-López et al. [33], studying finishing lambs for 42 d and supplemented with CaPr, did observe reductions in perirenal fat, coinciding with visceral organ mass remaining unaffected. Additionally, Carrillo-Muro et al. [9], who fed finishing lambs CaPr for 42 d, reported an increase in heart weight and small intestine weight, along with a tendency to increase liver mass, while other organ masses remained unaffected. Likewise, Macías-Cruz et al. [40] and Avendaño-Reyes et al. [41] did not detect effects of ZH on cattle organ weights. Nevertheless, Rivera-Villegas et al. [2] points out that ZH supplementation had a tendency of decreasing (8.9%) visceral fat, but with no effect on the mass of other organs. It is noteworthy that a decrease in hepatic weights has consistently been observed in feedlot lambs receiving ZH [29,30].

The supplementary energy provided by CaPr supplementation in the lamb diet contributes to the effectiveness of ZH, all while maintaining visceral organ mass. Rivera-Villegas et al. [2] proposed that the reductions in visceral organ mass contribute to the energy that is needed by the use of ZH. Moreover, Elam et al. [42] hypothesized that the increase in HCW and D% could be due to changes in visceral organ mass or a greater allocation of substrate to the carcass rather than the visceral organs.

### 4.4. Whole Cuts

Whole cuts are mainly affected by the nutritional level [43], resulting in varying rates of tissue growth and maturation, with higher levels seen in diets with abundant nutrition [44]. Similarly, Shadnoush et al. [45] indicate that a high energy level (2.64 Mcal/kg ME) in finishing lambs increases shoulder weight, while having no significant effect on the other whole cuts. They also note that as FBW increases, the weights of all cuts increase, except for the neck. These observations suggest that changes in feedlot lambs are the result of the increase in FBW caused by the increase in productive performance (ADG, DMI, and ADG:DMI ratio).

The results of this study reveal that the inclusion of CaPr when lambs are finished with ZH lead to increased values for breast IMPS209 and shoulder IMPS207, while other whole cuts were unaffected. Contrasting findings were reported by Carrillo-Muro et al. [10], who observed that in lambs supplemented with CaPr alone for varying durations, longer inclusion periods led to greater forequarter and neck weights at 28 d, whereas more extended inclusion periods reduced loin IMPS231 and increased the rack IMPS204. In contrast, Gomes et al. [46], in finishing lambs supplemented with glycerin, did not observe any effect on whole cuts.

Overall, in the present study, the inclusion of CaPr from 14 to 28 d alongside ZH supplementation led to a remarkable 19% increase in rack IMPS204 compared to ZH supplementation alone and a substantial 38% increase in comparison to CTL. The effect of ZH supplementation in increasing whole cuts is very common in feedlot cattle [47], with a more pronounced impact observed in the hindquarters [13]. However, there is no previous information on the effect of CaPr in the presence with ZH on whole cuts. Some studies indicate that ZH supplementation alone increases the loin IMPS231 and the leg IMPS233 [2,40]. Conversely, Avendaño-Reyes et al. [27] and Rojo-Rubio et al. [48] mention that ZH supplementation alone had no effect on the majority of whole cuts. The data from various authors exhibit considerable variability. As, such, Hilton et al. [49] suggest that the variation in the effect of ZH on whole cuts could depend on the presence or quantity of type II muscle fibers in each of these cuts, given that β2-adrenergic receptors are primarily located in these fibers.

### 4.5. Meat Characteristics

In the case of CKL%, the maximum value was estimated at 26 d of CaPr inclusion, which was lower compared to CTL and ZH. As the CaPr inclusion period increased, the PRL_24_% and PRL_48_% values rose, particularly with CaPr supplementation in conjunction with ZH. However, Carrillo-Muro et al. [10], in a study of fattening lambs supplemented with varying periods of CaPr alone, found that most of the meat characteristic variables were not affected. Nonetheless, they did observe an increase in muscle pH as the inclusion period of CaPr increased. However, Carrillo-Muro et al. [50] noted that ZH supplementation alone, when compared to CTL, does not appreciably affect WHC%, PRL%, color, or CKL%, but it led to a 36% increase in WBFS. Holmer et al. [15] also reported an 8% increase in CKL% in steers after 30 days of ZH supplementation.

In the present study, the inclusion of CaPr (14 to 28 d) in lambs fed ZH did not exhibit an effect on meat color. However, with regard to feedlot cattle, according to our study, the influence of ZH supplementation alone on color was negligible [42,51]. Contrary to this, Partida et al. [52] indicate that ZH supplementation alone in lambs either reduces, or tends to reduce, a* (redness). This phenomenon might arise from the dilution in myoglobin concentration resulting from an increase in sarcomere size due to ZH supplementation [53].

## 5. Conclusions

The gluconeogenic compound CaPr can be effectively incorporated in finishing diets for lambs undergoing supplementation with ZH during the final phase of fattening. When administered at a daily dosage of 10 g CaPr/lamb for 28 d, it significantly enhances the responses to ZH supplementation. This improvement is reflected in increased daily gain, enhanced feed efficiency, higher carcass weight, improved dressing percentage, and favorable alterations in the proportion and weight of certain whole cuts, all without adversely affecting carcass fat. However, it should be noted that CaPr supplementation does have a negative impact on CKL% and PRL% after 14 d of inclusion.

## Figures and Tables

**Table 1:** Ingredients of the basal diet offered to lambs and nutritional composition.

Ingredients	g kg^−1^ DM
Alfalfa hay mature	100.0
Oats hay	100.0
Dry-rolled corn yellow	500.0
Dried distiller grains	130.0
Soybean meal-44	54.0
Molasses cane	80.00
Calcium carbonate	10.0
Sodium bentonite	10.0
Sodium sesquicarbonate	15.0
Microminerals: Co, Fe, I, Mn, Zn, Se, and Cu ^a^	0.5
Vitamins: A, D and E ^b^	0.5
Chemical composition, g kg^−1^ DM ^c^	
Dry matter	835.1
Crude protein	137.2
Ether extract	23.8
Neutral detergent fiber	204.0
Calcium	8.5
Phosphorus	2.4
Ca:P ratio	3.5
Calculated net energy, Mcal/kg ^c^	
Maintenance	1.9
Gain	1.3

^a^ Microminerals: Co (0.5 g), Fe (50 g), I (2.5 g), Mn (50 g), Zn (50 g), Se (0.2 g) y Cu (15 g). Excipient q.s. 1000 g. ^b^ Vitamins A (5,000,000 IU), D (2,000,000 IU) y E (10,000 IU). Excipient q.s. 1000 g. ^c^ Based on the tabular values for individual feed ingredients (Ca, P, net energy for maintenance and gain) [17], with the exception of DM, CP, NDF, and EE (Ankom procedures) which were determined in our laboratory.

**Table 2:** Least square means, standard error of the mean (SEM), and orthogonal polynomials of productive performance, according to the duration of calcium propionate (CaPr) supplementation in lambs finished with zilpaterol hydrochloride (ZH).

Item ^b^	Control	Duration of CaPr Supplementation + ZH ^a^				SEM	Effects (*p-*Value)	
		0	14	28	42		Linear	Quadratic
IBW, kg	40.2	40.1	40.0	40.2	40.0	0.83	1.00	0.96
FBW, kg	49.4 ^b^	49.9 ^b^	51.2 ^ab^	54.1 ^a^	51.5 ^ab^	0.85	0.06	0.04
ADG, kg/d	0.220 ^b^	0.234 ^b^	0.263 ^ab^	0.333 ^a^	0.272 ^ab^	0.02	0.06	0.04
DMI, kg/d	1.4 ^a^	1.1 ^b^	1.3 ^a^	1.2 ^ab^	1.1 ^b^	0.06	0.33	0.15
ADG:DMI ratio	0.150 ^c^	0.204 ^b^	0.205 ^b^	0.264 ^a^	0.219 ^b^	0.01	0.07	0.09

^a^ Treatments consisted of the following: (1) No additives (CTL), (2) 0 days in CaPr plus 28 d on ZH, (3) 14 days in CaPr plus 28 d on ZH, (4) 28 days in CaPr plus 28 d on ZH, and (5) 42 days in CaPr plus 28 d on ZH. At the end of the fattening period, all lambs received a withdrawal period of 3 d before slaughter. CaPr was administered at a dose of 10 g/lamb/d and ZH at a dose of 7.2 mg/lamb/d. ^b^ IBW = Initial body weight, FBW = final body weight, ADG = average daily gain, DMI = dry matter intake. ^a,b,c^ Means a row with different superscripts differ (*p* < 0.05) according to Tukey’s test. FBW *y* = −0.0052*x*^2^ + 0.2639*x* + 49.545 (*R*² = 0.7286), maximal value at 26.4 d of inclusion for 53.0 kg. ADG *y* = −0.0001*x*^2^ + 0.0061*x* + 0.2254 (*R*² = 0.7154), maximal value at 30.5 d of inclusion for 0.318 kg/d.

**Table 3:** Least square means, standard error of the mean (SEM), and orthogonal polynomials of ultrasound measurements and carcass characteristics, according to the duration of calcium propionate (CaPr) supplementation in lambs finished with zilpaterol hydrochloride (ZH).

Item ^b^	Control	Duration of CaPr Supplementation + ZH ^a^				SEM	Effects (*p-*Value)	
		0	14	28	42		Linear	Quadratic
Ultrasound measurements								
Fat thickness, mm	2.9	3.1	4.0	3.4	3.8	0.32	0.90	0.20
LMA, cm^2^	12.4 ^b^	12.5 ^b^	13.8 ^a^	12.6 ^ab^	12.7 ^ab^	0.31	0.10	0.05
Carcass characteristics								
HCW, kg	23.8 ^b^	24.0 ^b^	25.8 ^ab^	26.3 ^a^	25.4 ^ab^	0.43	0.003	0.72
CCW, kg	22.9 ^b^	23.1 ^b^	25.0 ^ab^	25.6 ^a^	24.6 ^ab^	0.44	0.002	0.71
Dressing percentage	53.3 ^b^	55.8 ^b^	58.0 ^a^	58.0 ^a^	58.6 ^a^	0.45	0.0001	0.05
Cooling loss, %	3.2	3.5	3.2	2.4	3.1	0.46	0.22	0.50
Carcass length, cm	71.8	68.0	69.2	70.5	67.9	1.73	0.73	0.10
Leg circumference, cm	44.9	46.4	47.9	48.6	48.3	0.79	0.03	0.61
Chest circumference, cm	27.9	25.1	27.0	25.0	25.7	0.9	0.10	0.61
Shoulder composition								
Muscle, %	61.9	59.2	62.4	61.8	61.0	2.06	0.76	0.58
Fat, %	21.1	20.8	18.4	18.4	20.0	1.70	0.23	0.91
Bone, %	17.1	20.1	19.3	19.9	19.0	0.84	0.08	0.11

^a^ Treatments consisted of the following: (1) No additives (CTL), (2) 0 days in CaPr plus 28 d on ZH, (3) 14 days in CaPr plus 28 d on ZH, (4) 28 days in CaPr plus 28 d on ZH, and (5) 42 days in CaPr plus 28 d on ZH. At the end of the fattening period, all lambs received a withdrawal period of 3 d before slaughter. CaPr was administered at a dose of 10 g/lamb/d and ZH at a dose of 7.2 mg/lamb/d. ^b^ LMA= longissimus muscle area, HCW = hot carcass weight, CCW = cold carcass weight. ^a,b^ Means a row with different superscripts differ (*p* < 0.05) according to Tukey’s test. Dressing percentage *y* = −0.0032*x*^2^ + 0.2504*x* + 53.205 (*R*² = 0.988), maximal value at 39.1 d of inclusion for 58.1%.

**Table 4:** Least square means, standard error of the mean (SEM), and orthogonal polynomials of visceral organ mass, according to the duration of calcium propionate (CaPr) supplementation in lambs finished with zilpaterol hydrochloride (ZH).

Item ^b^	Control	Duration of CaPr Supplementation + ZH ^a^				SEM	Effects (*p-*Value)	
		0	14	28	42		Linear	Quadratic
Empty BW, kg	53.2 ^b^	56.2 ^b^	58.0 ^a^	57.7 ^a^	58.9 ^a^	0.60	0.01	0.03
Skin	148.6	135.1	151.5	160.1	160.6	13.10	0.43	0.32
Limbs	24.4	26.3	28.7	26.6	28.2	0.93	0.51	0.24
Head	39.1	37.9	41.6	41.1	40.9	1.34	0.11	0.75
Heart	4.9	4.6	5.5	5.3	6.1	0.40	0.40	0.83
Lungs	23.6	27.1	25.1	26.0	23.7	1.55	0.54	0.30
Liver	21.1	20.5	22.0	22.0	21.9	1.22	0.39	0.71
Spleen	2.0	2.0	1.9	2.0	2.2	0.30	0.91	0.84
Kidney	2.7	3.0	3.3	3.1	3.5	0.30	0.12	0.34
Testicles	15.4	14.1	15.6	15.4	14.7	1.10	0.83	0.50
Visceral fat	23.1	27.0	22.9	26.0	25.7	3.87	0.83	0.91
Perirenal fat	14.6	13.3	13.7	12.4	13.7	1.38	0.44	0.91
Stomach ^c^	29.9	27.3	29.7	25.3	26.1	1.82	0.23	0.63
Large intestine	10.6	11.4	12.0	9.5	10.2	1.27	0.63	0.14
Small intestine	18.3	17.0	20.5	21.6	19.8	1.63	0.12	0.43

^a^ Treatments consisted of the following: (1) No additives (CTL), (2) 0 days in CaPr plus 28 d on ZH, (3) 14 days in CaPr plus 28 d on ZH, (4) 28 days in CaPr plus 28 d on ZH, and (5) 42 days in CaPr plus 28 d on ZH. At the end of the fattening period, all lambs received a withdrawal period of 3 d before slaughter. CaPr was administered at a dose of 10 g/lamb/d and ZH at a dose of 7.2 mg/lamb/d. ^b^ Non carcass components are expressed in g/kg of empty body weight. ^c^ Includes the rumen-reticulum, omasum, and abomasum. ^a,b^ Means a row with different superscripts differ (*p* < 0.05) according to Tukey’s test. Empty BW *y* = −0.0008*x*^2^ + 0.0879*x* + 56.38 (*R*² = 0.8286), maximal value at 54.9 d of inclusion for 58.8 kg.

**Table 5:** Least square means, standard error of the mean (SEM), and orthogonal polynomials of whole cuts, according to the duration of calcium propionate (CaPr) supplementation in lambs finished with zilpaterol hydrochloride (ZH).

Item ^b^	Control	Duration of CaPr Supplementation + ZH ^a^				SEM	Effects (*p-*Value)	
		0	14	28	42		Linear	Quadratic
Whole cuts, g/kg of EBW								
Forequarter	6.2	6.1	6.4	6.7	6.1	0.16	0.11	0.32
Hindquarter	5.3	5.5	5.5	5.4	5.36	0.21	0.75	0.34
Shoulder IMPS207	2.1 ^b^	2.3 ^ab^	2.2 ^ab^	2.4 ^a^	2.2 ^ab^	0.06	0.09	0.63
Shoulder IMPS206	1.1	1.1	1.2	1.1	0.9	0.09	0.73	0.61
Leg IMPS233	3.0	3.1	3.2	3.0	3.1	0.10	0.83	0.14
Loin IMPS231	1.3	1.2	1.4	1.4	1.26	0.11	0.45	0.82
Rack IMPS204	0.77	0.62	0.76	0.73	0.58	0.03	0.92	0.21
Ribs IMPS209A	0.69	0.66	0.64	0.65	0.64	0.03	0.48	0.45
Flank IMPS232	0.91	1.1	0.9	0.99	0.96	0.04	0.82	0.64
Breast IMPS209	0.87 ^b^	0.93 ^b^	1.1 ^ab^	1.1 ^a^	1.0 ^ab^	0.03	0.004	0.40
Neck	0.70	0.47	0.62	0.69	0.68	0.11	0.81	0.13
Whole cuts, as percentage of CCW								
Forequarter	51.0	50.1	52.8	55.3	49.9	1.82	0.11	0.34
Hindquarter	43.3	45.1	45.1	44.3	44.0	1.5	0.75	0.33
Shoulder IMPS207	8.5	9.5	9.2	9.8	9.1	0.35	0.07	0.64
Shoulder IMPS206	4.4	4.5	4.8	4.5	3.7	0.45	0.75	0.61
Leg IMPS233	12.5	13.0	13.0	12.4	12.9	0.42	0.86	0.10
Loin IMPS231	5.4	5.1	6.0	5.7	5.2	0.43	0.45	0.90
Rack IMPS204	2.2 ^b^	2.5 ^b^	3.1 ^a^	3.0 ^a^	2.4 ^b^	0.21	0.94	0.13
Ribs IMPS209A	2.9	2.7	2.6	2.7	2.6	0.14	0.33	0.42
Flank IMPS232	3.7	4.4	3.6	4.1	4.0	0.25	0.74	0.61
Breast IMPS209	3.6	3.8	4.1	4.7	4.3	0.22	0.002	0.53
Neck	2.9	1.9	2.5	2.9	2.8	0.48	0.83	0.10

^a^ Treatments consisted of the following: (1) No additives (CTL), (2) 0 days in CaPr plus 28 d on ZH, (3) 14 days in CaPr plus 28 d on ZH, (4) 28 days in CaPr plus 28 d on ZH, and (5) 42 days in CaPr plus 28 d on ZH. At the end of the fattening period, all lambs received a withdrawal period of 3 d before slaughter. CaPr was administered at a dose of 10 g/lamb/d and ZH at a dose of 7.2 mg/lamb/d. ^b^ Whole cuts are expressed in g/kg of empty body weight (EBW) and percentage of cold carcass weight (CCW). ^a,b^ Means a row with different superscripts differ (*p* < 0.05) according to Tukey’s test.

**Table 6:** Least square means, standard error of the mean (SEM), and orthogonal polynomials of meat characteristics and meat color, according to the duration of calcium propionate (CaPr) supplementation in lambs finished with zilpaterol hydrochloride (ZH).

Item ^b^	Control	Duration of CaPr Supplementation + ZH ^a^				SEM	Effects (*p-*Value)	
		0	14	28	42		Linear	Quadratic
Meat characteristics								
pH_24 h_	5.3	5.4	5.7	5.7	5.58	0.19	0.08	0.7
Purge loss_24 h_, %	0.44	0.47	0.98	0.65	0.71	0.12	0.06	0.11
Purge loss_48 h_, %	0.41 ^b^	1.5 ^ab^	1.4 ^ab^	2.1 ^a^	1.9 ^a^	0.37	0.03	0.63
Cook loss, %	12.4 ^b^	12.2 ^b^	18.2 ^a^	19.1 ^a^	16.7 ^a^	2.17	0.14	0.001
WHC, %	11.4	13.8	14.5	14.4	14.0	2.17	0.30	0.54
WBSF, kg/cm^2^	4.2	4.0	3.9	3.9	4.2	0.1	0.28	0.74
Color								
L*	44.2	43.3	47.2	44.8	42.5	2.18	0.54	0.70
a*	16.9	16.1	13.5	16.0	16.3	1.23	0.13	0.72
b*	4.4	4.2	3.4	4.0	4.4	0.56	0.24	0.14

^a^ Treatments consisted of the following: (1) No additives (CTL), (2) 0 days in CaPr plus 28 d on ZH, (3) 14 days in CaPr plus 28 d on ZH, (4) 28 days in CaPr plus 28 d on ZH, and (5) 42 days in CaPr plus 28 d on ZH. At the end of the fattening period, all lambs received a withdrawal period of 3 d before slaughter. CaPr was administered at a dose of 10 g/lamb/d and ZH at a dose of 7.2 mg/lamb/d. ^b^ Meat characteristics were measured on the longissimus muscle between the 12th rib and the 2nd lumbar vertebrae. WHC = water-holding capacity, WBSF = Warner-Bratzler shear force. ^a,b^ Means a row with different superscripts differ (*p* < 0.05) according to Tukey’s test. Cook loss percent *y* = −0.0107*x*^2^ + 0.5529*x* + 12.29 (*R*² = 0.9942), maximal value at 25.8 d of inclusion for 19.4%.

## Data Availability

The information published in this study is available on request from the corresponding author.
